# Maitland mobilisation improves all ranges of motion in non-traumatic shoulder injury: A randomised controlled trial

**DOI:** 10.4102/sajp.v82i1.2325

**Published:** 2026-04-16

**Authors:** Karishma. C. Lalwani-Mangtani, Álvaro Ramos-Luzardo, Ricardo Chirino, Pedro Saavedra, Pilar Fernández-Valerón

**Affiliations:** 1 University of Las Palmas de Gran Canaria, Las Palmas de Gran Canaria, Spain; 2 Department of Rehabilitation and Biomechanics, ICOT Las Palmas Polyclinic Group, Las Palmas de Gran Canaria, Spain; 3 Fernando Pessoa Canarias University, Guía, Spain; 4 Department of Biochemistry and Molecular Biology, Physiology, Genetics and Immunology, University of Las Palmas de Gran Canaria, Las Palmas de Gran Canaria, Spain; 5 Department of Mathematics, University of Las Palmas de Gran Canaria, Las Palmas de Gran Canaria, Spain

**Keywords:** Maitland mobilisation, kinesiotherapy, shoulder rehabilitation, manual therapy, non-traumatic shoulder disorders

## Abstract

**Background:**

Shoulder pathologies are common causes of pain and functional limitation, often requiring physiotherapeutic interventions. Evidence comparing the effectiveness of manual therapy and exercise-based approaches in non-traumatic shoulder conditions remains limited.

**Objectives:**

To compare the effectiveness of Maitland mobilisation (MAIT) versus kinesiotherapy (KINE) in improving pain, function, quality of life, and range of motion (ROM) in patients with non-traumatic shoulder pathology.

**Method:**

Fifty-nine patients (63 shoulders) were randomly assigned to KINE (KINE; *n* = 32) or MAIT (MAIT; *n* = 31) over 15 sessions (three per week for 5 weeks). Both groups also received shortwave diathermy, transcutaneous electrical nerve stimulation, and exercises. Outcomes included the Disabilities for the Arm, Shoulder and Hand (DASH), American Shoulder and Elbow Surgeons (ASES) scale, Visual Analogue Scale (VAS) for pain, Short Form Health Survey, and ROM assessed with a Qualisys motion capture system. Assessments were performed at baseline, post-treatment, and 2-week follow-up.

**Results:**

While both groups showed significant improvements in pain, function, and quality of life after treatment, maintained at 2-week follow-up, MAIT also improved all outcome measures across all ROMs. Percentage changes from baseline were significantly greater in the MAIT group for most variables at both time points. A greater proportion of patients in the MAIT group exceeded the minimal clinically important difference (MCID) for DASH, ASES, and VAS.

**Conclusion:**

Although both approaches produced positive outcomes, MAIT demonstrated superior effectiveness compared with KINE.

**Clinical Implications:**

Maitland mobilisation may be recommended as a preferred intervention for non-traumatic shoulder pathology in clinical practice, offering broader improvements in function, pain, and mobility without additional treatment burden.

## Introduction

The shoulder joint complex (SJC), a structurally intricate joint, provides articulation between the upper extremities and the axial skeleton. Owing to its unique anatomical configuration, the SJC has the maximum range of motion (ROM) among all joints in the human body. However, poor joint congruency, along with the demands of strength, stability, and flexibility placed on the SJC in everyday tasks, makes it particularly susceptible to musculoskeletal disorders (Chang, Anand & Varacallo 2025). Shoulder joint complex disorders constitute a very frequent problem in the general population, with an estimated prevalence ranging from 0.7% to 55.2%, being higher in women than in men and especially prevalent in high-income countries (Lucas et al. 2022). Symptoms can be persistent and disabling, leading to pain, functional limitations, and reduced quality of life, and interfering with daily activities at home and work, with significant economic costs in terms of increased healthcare demands and impaired work (Espahbodi et al. 2017).

Several overlapping medical conditions can lead to painful shoulders, including rotator cuff disorders, biceps tendinopathy, acromioclavicular joint abnormalities and adhesive capsulitis (Linaker & Walker-Bone 2015). A wide range of treatments has been proposed, including joint rest, pharmacological treatment with anti-inflammatory or analgesic drugs, active and passive mobilisation, electrotherapy, capsular distension, and manipulation under anaesthesia or arthroscopy (Crookes et al. 2023; Lucas et al. 2022).

Physiotherapeutic interventions focused on restoring joint mobility and function occupy a central role among numerous therapeutic strategies available for painful shoulder conditions. Manual therapy-based techniques aim to relieve pain and improve ROM through specific mobilisation procedures, while exercise-based techniques emphasise restoring muscular strength, endurance and motor control. Within this spectrum, two commonly employed interventions are Maitland mobilisation (MAIT), a structured manual therapy method based on graded oscillatory movements (Yaver 2007), and kinesiotherapy (KINE), a method that employs active and passive exercises to optimise recovery of shoulder function (Ansari, Mohammad Rahimi & Aminzadeh 2025).

Maitland mobilisation represents a structured and evidence-informed manual therapy approach, integrating clinical reasoning with graded passive movements to the spinal and peripheral joints. Based on the patient’s presentation, oscillatory techniques are applied at varying amplitudes and ranges. These techniques are classified into grades I to V, with grade V indicating the option of a small-amplitude, high-velocity thrust. Lower-grade techniques primarily address pain modulation through neurophysiological mechanisms, whereas higher-grade techniques reduce stiffness and restore mobility by targeting end-range restrictions, with the goal of increasing ROM and decreasing pain (Gutierrez-Espinoza et al. 2023). However, the efficacy of the MAIT method in painful shoulder pathologies remains poorly understood (Do Moon et al. 2015; Haider et al. 2018; Piekarz & Perry 2016).

Kinesiotherapy involves movement of some parts of the body or the whole body using exercises to maintain, establish, develop and change the functions of the locomotor apparatus and organs of locomotion. Kinesiotherapy aims to achieve optimal recovery of damaged locomotor function by using all the potential of the patient undergoing treatment. Kinesiotherapy includes both active and passive exercises. Active exercises are divided into those with assistance, without assistance, and with resistance (Ansari et al. 2025).

Recent evidence suggests that a therapeutic exercise constitutes a central element in managing shoulder pain, with additional modalities such as laser therapy demonstrating meaningful benefits. Manual therapy and educational interventions may also contribute to symptom improvement, although their effects appear less consistent and generally inferior to exercise-based interventions (Aguilar Garcia et al. 2024). Heat therapy, often delivered through shortwave diathermy (SWD), is well established to enhance multimodal treatment outcomes by increasing blood flow and metabolic activity and promoting pain relief, and it is therefore frequently combined with other physiotherapeutic interventions to mitigate shoulder pain and functional limitations (Sung, Lee & Kim 2022; Zanoli et al. 2024). Emerging evidence also suggests a likely contribution to skeletal muscle mass gain, although current findings remain preliminary (Rodrigues et al. 2020). Alongside these treatment strategies, transcutaneous electrical nerve stimulation (TENS) is commonly employed as a non-invasive analgesic tool to reduce symptomatic pain (Johnson et al. 2022).

Despite wide clinical use of both MAIT and KINE, their relative effectiveness within the context of multimodal shoulder rehabilitation remains poorly established. Addressing this gap is essential for guiding evidence-based decision-making in non-traumatic shoulder disorders. Therefore, we aimed to compare the effectiveness of these two commonly implemented approaches as an adjunctive treatment to TENS, SWD, and therapeutic exercise in the rehabilitation of painful shoulders, to determine which intervention yields greater improvements in pain, function, and shoulder mobility.

## Research methods and design

### Participants

Eligible participants were previously diagnosed with diverse shoulder pathologies by a traumatologist, who requested magnetic resonance imaging to make an accurate diagnosis and prescribed shoulder rehabilitation. Our study participants were consecutively recruited from September 2020 to October 2021 at ICOT Policlínico Las Palmas rehabilitation centre, Las Palmas de Gran Canaria, Spain.

Eligibility criteria required participants to present at least two limitations of shoulder ROM, attributable to either stiffness or pain, affecting movement in different anatomical axes. Exclusion criteria included shoulder pain secondary to cervical spine pathology; prior shoulder surgery; diabetes mellitus; local infection; severe trauma with fracture; pregnancy; malignancy; severe cardiac or psychiatric conditions; and mobility impairments associated with neurological disorders such as stroke or Parkinson’s disease, to prevent confounding influences arising from increased muscle tone or rigidity. Patients with rheumatologic disorders or osteoporosis were also excluded from participation (Ali & Khan 2015; Do Moon et al. 2015). Overall, 59 patients corresponding to 63 affected shoulders met the eligibility criteria and were included in our study. They reported pain lasting between 2 months and 6 months before the consultation.

### Sample size calculation

Given the limited literature directly comparing MAIT and KINE in shoulder pathologies, the sample size was determined using a pilot study, published in the *II Ibero-American Interdisciplinary Virtual Congress of Nursing and Physiotherapy* (ISBN: 978-84-16679-16-4); the pilot study had 10 subjects treated with MAIT and 10 controls treated with KINE. The reduction in the Disabilities for the Arm, Shoulder, and Hand (DASH) score was selected as the primary outcome, while improvements in the other scales and the various ROMs were defined as secondary outcomes. From the pilot study data, the DASH scale, with a mean of –64.5 for the experimental group and –34.0 for the control group, was considered the primary endpoint, with a common standard deviation (s.d.) of 32.6. For the observed difference, the *t*-test with a statistical significance of 5% achieved a power of 95%, with a sample size of 31 shoulders per treatment.

### Patient allocation

The enrolled patients were randomly allocated at a 1:1 ratio to receive in all the sessions either KINE (KINE group; *n* = 32 shoulders) or MAIT (MAIT group; *n* = 31 shoulders). Randomisation was done by the author Ricardo Chirino who was not involved in any treatment delivery, using the web-based randomisation system RANDOM.ORG (Randomness and Integrity Services Ltd, Dublin, Ireland; retrieved from https://www.random.org), which generates true random numbers from atmospheric noise.

To ensure confidentiality and anonymity, patients’ personal data were coded and made accessible only to the therapist responsible for administering the interventions.

### Interventions

Interventions and assessments were conducted by the same physical therapist (Karishma C. Lalwani-Mangtani), who possessed 3 years of experience in manual therapy and the management of musculoskeletal pain disorders at the time of patient recruitment.

The KINE group underwent assisted KINE aimed at targeting the full range of shoulder motion in supine, prone, and lateral decubitus and side positions. Movement in these positions aimed to optimise joint function by targeting capsule-ligamentous and muscular stretching. First of all, passive or assisted ROM exercises were undertaken to maintain or restore SJC mobility without imposing significant muscular activation. As patients’ symptoms improved, the intervention progressed to active assistive and fully active exercises, thereby facilitating greater motor control and promoting active engagement of the SJC musculature (Uhl, Muir & Lawson 2010).

Kinesiotherapy parameters were individualised according to each patient’s symptom irritability, pain level, and tolerance to shoulder movement. Progression to active-assisted and subsequently active exercises was permitted only when patients demonstrated decreased pain, improved movement quality, and adequate control throughout the available ROM. Patients who continued to exhibit high irritability, guarding, or pain exacerbation remained at the passive- or active-assisted level until clinical signs improved. Although the overall exercise structure was standardised, the intensity, level of assistance, and progression of each KINE component were individually adjusted based on each patient’s daily presentation.

In contrast, the MAIT group received Maitland accessory glide mobilisations in the supine position. Mobilisations were delivered in series at frequencies of 2 Hz – 3 Hz, each mobilisation set lasting 3 min with 1-min rest intervals between series (Piekarz & Perry 2016). For participants presenting with bilateral shoulder pathology (*n* = 4), the same treatment protocol was applied equally to both shoulders.

Glenohumeral accessory mobilisations were started with Grades I and II oscillations, which involve gentle, small-amplitude movements. In patients presenting with high pain levels or marked irritability, progression to higher grades was deferred until symptoms improved and pain abated. Subsequently, the intervention progressed to Grades III and IV mobilisations, which involve larger-amplitude movements aimed at enhancing joint mobility once pain allowed. Concerning the direction of mobilisation, anteroposterior glides were applied during shoulder flexion and extension, longitudinal glides during abduction, and posterior mobilisations were used to improve rotational movements.

Mobilisation grades were selected individually for each participant based on irritability and pain response. All patients initially received Grade I–II oscillatory mobilisations to minimise discomfort and modulate pain. Progression to Grade III–IV was permitted only when patients demonstrated reduced irritability, lower resting pain, and adequate tolerance to end-range passive movement. Patients with persistent high irritability or elevated pain levels were maintained exclusively on Grade I–II techniques throughout the session or across the treatment period. Thus, despite a standardised treatment regimen, the mobilisation grade applied to each patient was individualised according to their daily clinical presentation.

In both treatment groups, progression criteria were applied between sessions rather than within the same-treatment session. When patients tolerated the intervention grade delivered during a session, no further advancement was made during that visit; instead, progression to active assistive movement (KINE) or to Grade III–IV mobilisations (MAIT) was introduced at the next scheduled session.

All participants received TENS, SWD, and therapeutic exercises during each treatment session, besides KINE or MAIT. For TENS, electrodes were placed on the anterior and posterior aspects of the shoulder and then applied in continuous mode for 15 min. The stimulation protocol consisted of a frequency of 100 Hz, a pulse width of 250 microseconds (*µ*s), and an intensity sufficient to produce strong, non-painful paraesthesia beneath the electrodes (Johnson & Martinson 2007). For SWD application, electrodes were positioned with one plate anteriorly and the other posteriorly on the shoulder. Treatment was administered for 10 min in continuous mode at a carrier frequency of 27.12 MHz, with intensity individually adjusted to evoke a sensation of deep heat without discomfort (Robertson & Baker 2001).

Therapeutic exercise was progressively integrated throughout the treatment protocol. During the initial seven sessions, the participants performed several joint mobility exercises using a therapy stick to facilitate flexion, extension, abduction, adduction, internal rotation, and external rotation. Each session comprised five sets of 20 repetitions of each movement, emphasising controlled, pain-free motion to maximise joint flexibility (Mohamed et al. 2020). Beginning with the eighth session, concentric and eccentric strengthening of the rotator cuff was introduced to promote muscle strengthening and enhance functional stability. These strengthening exercises consisted of three sets of 15 repetitions and were progressively intensified under physiotherapist supervision to ensure correct technique and gradual overload, thereby supporting safe progression towards improved shoulder strength and functional outcomes (Kinsella et al. 2017).

### Outcome measures

The participants were evaluated at three distinct time points: at baseline (before the first treatment session), immediately following the final treatment session (first control), and 2 weeks after completion of treatment (second control). This 2-week post-treatment assessment was done to determine whether early improvements persisted shortly after the completion of therapy and to inform the need for subsequent intervention, if required.

Functional outcomes were assessed with the DASH questionnaire and the American Shoulder and Elbow Surgeons (ASES) scale. The DASH is a 30-item instrument specifically designed to measure symptoms and physical function in musculoskeletal disorders of the upper extremities. Scores range from 0 to 100, with higher values reflecting greater disability and reduced functional capacity (Beaton et al. 2001; Hudak et al. 1996). The ASES scale comprises 11 items distributed across two domains: pain (one item) and function (10 items). Each domain is scored from 0 to 50, and the total score (0–100) is obtained by summing scores of both domains, with higher scores indicating superior health status and improved shoulder function (Richards et al. 1994; Vrotsou et al. 2019). Pain intensity was measured using the Visual Analogue Scale (VAS), which rated participants’ pain on a scale from 0 (no pain) to 10 (worst imaginable pain). Visual Analogue Scale has shown strong reliability and validity in musculoskeletal pain assessment (Downie et al. 1978; Fillingim et al. 2016). General health and quality of life were measured using the Short Form Health Survey (SF-36), a 36-item questionnaire evaluating eight dimensions: vitality, physical functioning, bodily pain, general health, physical role functioning, emotional role functioning, social functioning, and mental health. Each domain yields a score between 0 and 100, where lower scores denote poorer health status and higher scores reflect better quality of life (Alonso, Prieto & Anto 1995; Ware & Sherbourne 1992).

Shoulder ROM was measured using a Qualisys Track Manager motion capture system, version 2.8 (Qualisys AB, Gothenburg, Sweden) equipped with nine high-resolution cameras. Reflective markers were placed on anatomical landmarks of the shoulder girdle, and the cameras captured three-dimensional movement patterns, which were subsequently processed with the Qualisys software. This approach provides real-time visualisation, biomechanical analysis, and data export, ensuring precise and reproducible measurement of kinematic variables (Almeida et al. 2023). Range of motion testing was conducted in the seated position and included the following movements: flexion, extension, abduction, adduction, and internal and external rotation. An improvement in mobility was defined as an increase in the degrees of motion across all planes, except in adduction, where a smaller angle denotes greater approximation to the neutral axis and is therefore interpreted as an improvement in shoulder function. The generated report from the Qualisys system provided both numerical and graphical feedback on shoulder kinematics, thereby allowing for detailed monitoring of treatment effects on joint mobility.

The primary outcome of the current study was defined as the percentage change in DASH score between baseline and the first control assessment. Functional recovery was prioritised as the principal endpoint given its direct relevance to the ability of patients to perform daily activities. Improvements in shoulder ROM, pain intensity as measured by the VAS, self-reported health status via the ASES score, and quality of life captured by the SF-36 were considered secondary outcomes.

### Data analysis

The data were summarised using appropriate descriptive statistical analysis according to the nature and distribution of each variable. Categorical variables were presented as absolute frequencies and percentages. For continuous variables, mean and s.d. values were reported for normal distribution, whereas median and interquartile range (IQR = 25th–75th percentile) were determined when normality assumptions were not satisfied. Normality was assessed using standard statistical criteria. Because of nonconformance of the majority of the variables to a Gaussian distribution, non-parametric tests were applied. Specifically, medians across more than two repeated measurements were compared using the Friedman test for related samples, which accounts for within-subject dependence in longitudinal data. When global differences were identified, post-hoc analyses were performed using the Durbin–Conover test to enable multiple pairwise comparisons while controlling for familywise error rates. This sequential procedure ensured robust identification of changes across baseline, post-treatment and follow-up assessments, even in the presence of non-normal data distributions.

To evaluate the relative efficacy of the two intervention groups and identify which method produced superior outcomes, additional analyses were used to determine percentage changes in each outcome variable relative to baseline. Two new variables were derived: improvement at the end of treatment; first control = (post treatment – baseline)/baseline × 100 and improvement at follow-up; second control = (follow-up – baseline)/baseline × 100. These relative improvement indices enabled direct comparison of treatment responsiveness between groups. Group comparisons of the mean improvement vectors employed a multivariate Wald-type test, with probability distributions under the null hypothesis approximated by bootstrap resampling (Konietschke et al. 2015). When multiple group contrasts were required, the Games–Howell procedure was applied as it does not assume homogeneity of variances and is appropriate for unequal sample sizes (Games & Howell 1976). Effect size for significant between-group differences was measured by Cliff’s delta (a test for non-parametric distributions).

Finally, absolute change scores were determined for DASH, ASES, and VAS scores at post-treatment (*first control – baseline*) and follow-up (*second control – baseline*). The change scores were dichotomised depending on their meeting the MCID for each scale. Based on established values for shoulder rehabilitation, MCIDs were defined as 10.83 points for DASH (Franchignoni et al. 2014), 12 points for ASES (Tashjian et al. 2010), and 3 cm for VAS (Tashjian et al. 2009). Between-group comparisons of the proportion of patients achieving the MCID were evaluated using Chi-square (*χ*^2^) tests.

All statistical analyses were performed using R software, version 4.2.1 (R Development Core Team, 2022). Statistical significance level was set at a two-tailed *p* value < 0.05 for all comparisons.

## Results

Our study cohort comprised 34 women and 25 men aged 23–83 years (mean 55.6 ± 14.8 years). No significant differences were observed between the two groups in terms of age distribution, sex ratio or frequency of specific shoulder pathologies, indicating adequate sample comparability. Across both groups, rotator cuff tendinosis was the most frequently diagnosed condition, followed by other common non-traumatic shoulder pathologies (Table 1). These findings suggest that the allocation of patients resulted in two balanced cohorts, thereby allowing subsequent comparisons between the intervention groups to be interpreted with greater validity.

**TABLE 1:** Baseline characteristics of the patients enrolled in the study.

Variable	Total (*N* = 63)			KINE (*n* = 32)			MAIT (*n* = 31)		
	*n*	%	Mean ± s.d.	*n*	%	Mean ± s.d.	*n*	%	Mean ± s.d.
Age† (years)	-	-	55.6 ± 14.8	-	-	56.1 ± 16.9	-	-	55.2 ± 12.6
**Sex†**									
Women	34	57.6	-	14	48.3	-	20	66.7	-
Men	25	42.4	-	15	51.7	-	10	33.3	-
**Main diagnosis**									
Rotator cuff tendinosis	44	69.8	-	22	68.8	-	22	71.0	-
Rotator cuff tendinosis and subacromial bursitis	11	17.4	-	5	15.6	-	6	19.4	-
Rotator cuff tendinosis and adhesive capsulitis	1	1.6	-	0	0.0	-	1	3.2	-
Subacromial syndrome	3	4.8	-	2	6.3	-	1	3.2	-
Bursitis and subacromial syndrome	2	3.2	-	1	3.1	-	1	3.2	-
Bicipital tendinosis	2	3.2	-	2	6.3	-	0	0.0	-

KINE, Kinesiotherapy; MAIT, Maitland mobilisation; s.d., standard deviation.

† , Data from 59 patients.

The current study aimed primarily to evaluate the effectiveness of MAIT and KINE in improving joint mobility and quality of life. Therefore, participants were assessed at baseline, after 5 weeks of treatment (first control), and at a 2-week follow-up after the completion of treatment (second control), using ROM measures and the DASH, ASES, VAS and SF-36 scales. In the KINE group, remarkable improvements in flexion, abduction and external rotation ROMs, and in all four clinical scales (DASH, ASES, VAS and SF-36) post-treatment (Table 2). These gains were generally maintained at the 2-week follow-up, although flexion improvement lost statistical significance. In contrast, the MAIT group exhibited significant improvements in all ROMs and all clinical scales both at both post-treatment and follow-up, demonstrating more consistent and sustained effects of these interventions.

**TABLE 2:** Effects of kinesiotherapy and Maitland mobilisation on the variables analysed.

Variable	Treatment	Baseline		First control		Second control		*P*-value†	*P*-value‡	*P*-value§
		Mean	IQR	Mean	IQR	Mean	IQR			
DASH	KINE	35.0	23.4 ; 53.8	23.0	12.3 ; 41.7	22.6	9.3 ; 47.3	< 0.001	< 0.001	< 0.001
	MAIT	37.5	23.1 ; 52.5	16.9	6.6 ; 34.0	11.3	4.5 ; 26.3	< 0.001	< 0.001	< 0.001
ASES	KINE	44.2	26.3 ; 55.0	56.7	31.2 ; 81.7	57.8	30.1 ; 86.4	0.002	0.004	< 0.001
	MAIT	38.3	26.7 ; 49.2	70.0	53.0 ; 80.0	75.0	57.5 ; 85.8	< 0.001	< 0.001	< 0.001
VAS	KINE	7.0	6.0 ; 9.0	5.5	2.0 ; 7.0	5.0	1.0 ; 7.0	< 0.001	< 0.001	< 0.001
	MAIT	7.0	6.0 ; 8.5	4.0	3.0 ; 5.0	3.0	1.5 ; 5.0	< 0.001	< 0.001	< 0.001
SF-36	KINE	75.9	56.6 ; 80.4	76.2	63.0 ; 82.6	76.5	60.0 ; 84.6	< 0.001	< 0.001	< 0.001
	MAIT	75.3	67.3 ; 78.6	79.1	74.6 ; 87.5	85.5	77.2 ; 89.2	< 0.001	< 0.001	< 0.001
FLX	KINE	145.1	107.0 ; 166.6	154.0	123.0 ; 167.0	148.0	127.0 ; 165.0	0.007	0.001	0.098
	MAIT	140.0	122.5 ; 150.9	156.0	143.0 ; 165.0	163.0	151.0 ; 171.0	< 0.001	< 0.001	< 0.001
EXT	KINE	32.0	19.1 ; 48.1	39.2	24.9 ; 53.8	33.1	24.7 ; 44.6	0.395	NA	NA
	MAIT	31.7	21.9 ; 42.3	46.6	32.3 ; 53.9	55.6	37.2 ; 59.7	< 0.001	< 0.001	< 0.001
ABD	KINE	124.4	78.7 ; 150.3	133.0	107.0 ; 156.0	131.0	87.5 ; 158.0	< 0.001	0.002	0.001
	MAIT	110.0	95.6 ; 133.3	139.0	122.0 ; 151.0	142.0	133.0 ; 157.0	< 0.001	< 0.001	< 0.001
ADD	KINE	10.4	4.2 ; 16.2	7.0	2.0 ; 12.1	7.2	4.0 ; 11.6	0.155	NA	NA
	MAIT	5.9	3.5 ; 12.7	3.9	2.1 ; 7.4	2.5	1.3 ; 4.6	< 0.001	< 0.001	< 0.001
IR	KINE	84.0	69.2 ; 90.9	88.7	73.5 ; 96.2	88.6	72.5 ; 94.5	0.168	NA	NA
	MAIT	78.5	73.5 ; 93.5	92.5	78.2 ; 100.0	96.8	83.9 ; 105.0	< 0.001	< 0.001	<0.001
ER	KINE	36.5	22.6 ; 51.7	46.4	21.8 ; 61.2	49.5	21.4 ; 63.9	< 0.001	< 0.001	< 0.001
	MAIT	28.7	20.1 ; 46.9	52.9	39.2 ; 62.6	58.2	48.6 ; 76.0	< 0.001	< 0.001	< 0.001

Note: Data are expressed as median (interquartile range [IQR]). Baseline: prior to treatment; First control: at the end of treatment; Second control: after 2 weeks of follow-up.

KINE, Kinesiotherapy; MAIT, Maitland method; DASH, Disabilities of the Arm, Shoulder, and Hand scale; ASES, American Shoulder and Elbow Surgeons scale; VAS, Visual Analogue Scale; SF-36, Short Form Health Survey; FLX, Flexion; EXT, extension; ABD, Abduction; ADD, Adduction; IR, internal rotation; ER, external rotation; NA, not applicable.

† , Friedman test for related samples.

‡ , Post-hoc Durbin-Conover test for pairwise comparisons (Baseline vs. First control).

§ , Post-hoc Durbin-Conover test for pairwise comparisons (Baseline vs. Second control). Not applicable values denote that post-hoc Durbin-Conover testing was not warranted because of non-significant Friedman test outcomes.

**FIGURE 1:**
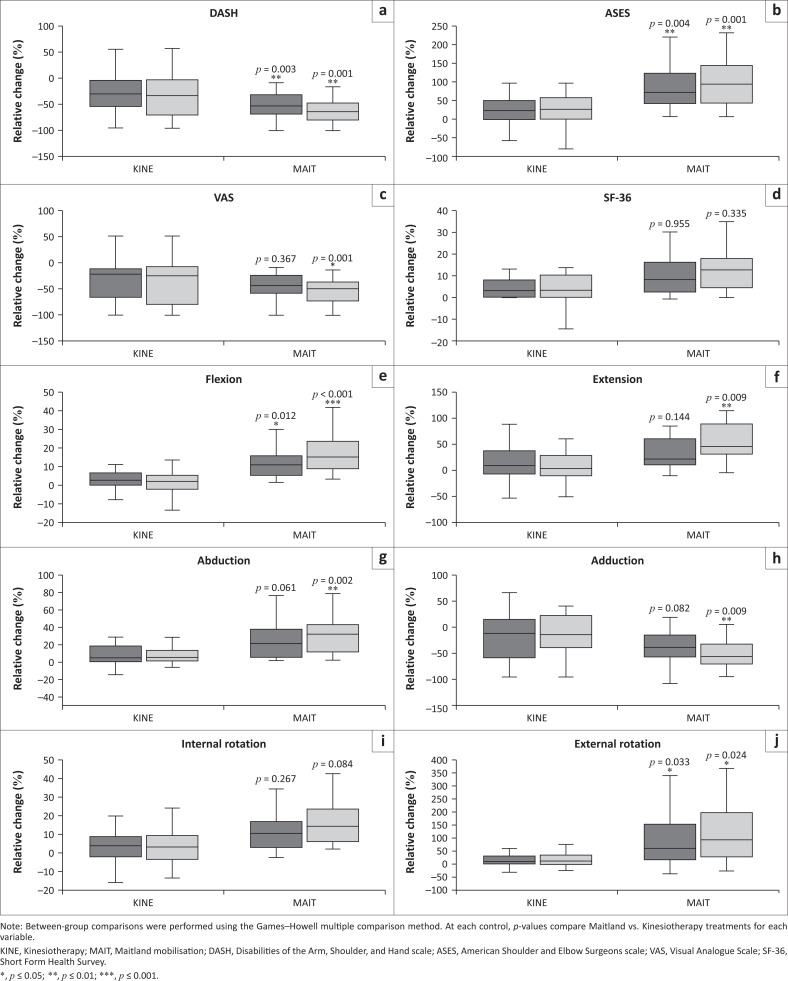
Percentage change from baseline in shoulder function and range of motion (ROM) at post-treatment (first control) and follow-up (second control) in the Maitland mobilisation (MAIT) and kinesiotherapy (KINE) groups, represented as box plots. Dark grey boxes correspond to post-treatment values (first control), and light grey boxes correspond to follow-up values (second control). (a) DASH, (b) ASES, (c) VAS, (d) SF-36, (e) flexion, (f) extension, (g) abduction, (h) adduction, (i) internal rotation and (j) external rotation.

After confirming that both MAIT and KINE groups supported functional recovery, their relative efficacy was directly compared. For this purpose, percentage changes relative to baseline were calculated for each outcome variable at the two assessment points, as described in the statistical analysis. Results reveal key between-groups differences. At the first control, the MAIT group showed significantly greater improvements than KINE in flexion, external rotation, DASH and ASES scores (Figure 1; Online Appendix 1 – Table 1). Furthermore, at the second control, the superiority of MAIT became more pronounced with significantly better results observed across nearly all variables, including ROM and clinical scales, except internal rotation and SF-36. The Cliff’s δ effect size estimates further reinforce these findings (Online Appendix 2 – Table 2), suggesting consistent differences between groups.

**FIGURE 2:**
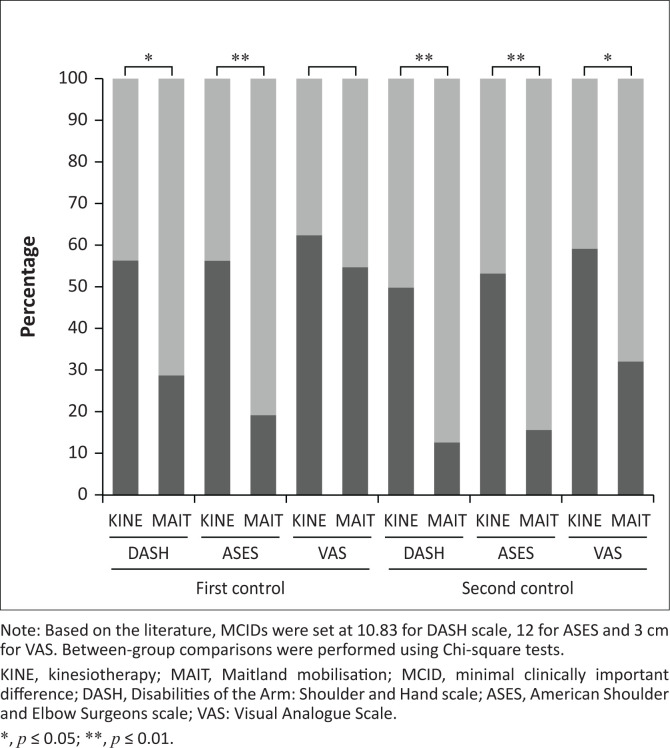
Proportion of patients achieving clinically meaningful improvement (≥ MCID) in the Maitland mobilisation (MAIT) and kinesiotherapy (KINE) groups at post-treatment (first control) and follow-up (second control), represented as stacked column charts. Light grey indicates the percentage of patients whose change exceeded the minimal clinically important difference (≥ MCID), whereas dark grey represents changes below the MCID (< MCID).

Absolute change scores were calculated for DASH, ASES, and VAS at post-treatment and follow-up and were compared with their established MCIDs (10.83 for DASH, 12 for ASES, and 3 cm for VAS) to evaluate the proportion of patients achieving clinically meaningful improvement. Proportion analyses revealed that, at post-treatment, significantly more patients in the MAIT group met the MCID thresholds for DASH and ASES, whereas no between-group differences were observed for VAS. At follow-up, and in line with the patterns observed in previous analyses, MAIT achieved a higher MCIDs on all three scales at follow-up (Figure 2; Online Appendix 3 – Table 3). Overall, these findings indicate that, while both treatment modalities are beneficial, MAIT provides broader, more sustained and clinically meaningful improvements in shoulder mobility, pain, and function compared with KINE.

## Discussion and conclusion

Our study provides novel evidence that MAIT represents a valuable therapeutic alternative for patients with non-traumatic shoulder pathology. The global expansion of computer use may contribute to the rising prevalence of arm, neck, and shoulder disorders. Painful shoulder conditions frequently cause disability worldwide, substantial health care utilisation, prolonged sick leave and decreased quality of life (Kang et al. 2024). Resultantly, identifying cost-effective, clinically applicable and sustainable interventions remains a priority in musculoskeletal rehabilitation.

Both interventions proved effective in improving pain, shoulder function, and quality of life, validating their therapeutic relevance in non-traumatic shoulder rehabilitation. However, participants treated with MAIT demonstrated significantly better outcomes post-treatment, and these improvements were not only maintained but, in most cases, enhanced at follow-up (Figure 1). Notably, the fact that most patients who underwent MAIT exceeded the MCID thresholds across all functional and pain-related scales highlights the clinical relevance of these improvements (Figure 2). From a clinical perspective, these findings suggest the potential for sustained benefit and support incorporating MAIT into routine management strategies, although future studies with extended follow-up are needed to determine whether these effects persist over time and confirm its longer-term superiority.

To date, no previous study has directly compared MAIT with KINE. Furthermore, the evidence supporting KINE for conservative shoulder rehabilitation remains scarce. Sumariva-Mateos et al. (2022) compared KINE with myofascial techniques in 44 patients with immobilised shoulders and found improvements in function, mobility, and pain in both groups, though clinically meaningful pain reduction was achieved only with myofascial therapy (Sumariva-Mateos et al. 2022). This suggests that although KINE is beneficial for shoulder rehabilitation, its effect may be less consistent in shoulder pathology than other approaches.

The body of evidence for MAIT in shoulder rehabilitation is limited but promising. Another study compared the Maitland method with the Kaltenborn technique in 20 patients with frozen shoulder, reporting significant improvements in both groups without between-group differences (Do Moon et al. 2015). However, the exclusive focus on frozen shoulder limits the generalisability of those results to other pathologies.

Haider et al. demonstrated that the Maitland technique combined with conservative exercise was superior to exercise alone for patients with subacromial syndrome, yielding significant reductions in pain and improved function and mobility (Haider et al. 2018). Importantly, however, in that trial, MAIT was applied to the thoracic region rather than directly to the shoulder, which may have influenced the results. A similar approach was adopted by Elgendy et al., who compared conventional exercise with Maitland and Mulligan thoracic mobilisations in men with subacromial impingement syndrome, reporting improvements across all interventions. However, Mulligan mobilisation achieved the greatest gains in pain reduction, abduction and external rotation ROM, and kyphosis correction (Elgendy et al. 2024). Together, these studies suggest that spinal manual therapy may contribute to symptomatic improvement in shoulder disorders, although the indirect application limits conclusions regarding its specific efficacy at the shoulder joint. The current research extends this evidence by demonstrating the efficacy of MAIT when applied directly to the shoulder in a broader population with heterogeneous non-traumatic shoulder pathologies.

The present study results align with those of previous studies in evaluating the effectiveness of MAIT when applied directly to the shoulder joint. Celik and Menek (2025) conducted a study of 45 individuals with rotator cuff lesions, showing that both Mulligan and MAITs produced greater improvements in pain, function, and ROM than conventional exercise, with Mulligan yielding the most pronounced gains in quality-of-life domains (Celik & Menek 2025). Additionally, Khandaloo et al. investigated the effects of Mulligan and Maitland shoulder mobilisation techniques combined with contemporary physical therapy on acromiohumeral distance in 51 overhead athletes with primary subacromial impingement syndrome. Both techniques significantly increased acromio-humeral distance at three static angles of passive scapular abduction, while no changes were observed in the control group. The Mulligan approach produced greater improvements than the Maitland technique, suggesting that both mobilisations are effective adjuncts to physical therapy, with Mulligan providing superior outcomes (Khandaloo et al. 2025).

Maitland mobilisation has also been compared with other techniques. A randomised controlled trial investigated the effects of muscle energy technique (MET) and MAIT on shoulder pain and disability in 30 patients following neck dissection surgery. Both interventions, combined with conventional physiotherapy, significantly improved pain, disability, and shoulder ROM. However, MET demonstrated superior efficacy compared to MAIT, suggesting that MET is a more effective approach for restoring shoulder function and quality of life in the postoperative period (Elhameed et al. 2024).

Lastly, Singh et al. compared the immediate effects of kinesio taping (K-taping) and Grade IV MAIT on glenohumeral internal rotation deficit in 32 asymptomatic overhead athletes. Even though both interventions produced significant improvements in internal rotation, no differences were detected between groups or among different sports. These findings indicate that K-taping is equally effective for enhancing shoulder internal rotation in overhead athletes with glenohumeral internal rotation deficit (Singh et al. 2023).

Collectively, the extant literature supports the effectiveness of different mobilisation techniques, such as Maitland, Mulligan, and MET in improving shoulder pain, function, and ROM across various populations. While Mulligan mobilisation demonstrates superior outcomes compared to other techniques and MET shows promise, more studies involving larger, more heterogeneous cohorts with comprehensive patient data are required to elucidate the specific clinical scenarios to optimally indicate each technique.

Our study has important methodological strengths. The interventions were delivered in a standardised manner; outcomes were assessed with validated scales and objective kinematic measures; and our study design included both post-treatment and short-term follow-up assessments. Moreover, incorporating MCID thresholds also strengthens the clinical relevance of the results, helping to contextualise the observed changes beyond statistical significance. Such a multidimensional evaluation supports the robustness of our findings and enhances their clinical interpretability.

Our study suffers from some limitations. Firstly, the wide age range of the participants may have introduced variability in treatment response, as younger and older patients may differ in tissue elasticity, recovery potential, and adherence. Secondly, the inclusion of a heterogeneous sample of shoulder pathologies could have impacted outcome variability. However, the absence of significant differences in baseline age or diagnosis distribution between groups alleviates concerns about bias. Thirdly, the follow-up period was limited to 2 weeks, precluding conclusions about long-term effects. Fourthly, observer and performance bias might have been introduced as the same physiotherapist administered all interventions and performed all assessments. Finally, although the sample size was sufficient to detect differences between groups, larger multicentre trials are needed to confirm and extend our findings.

Future research should address these limitations by focusing on specific pathologies, stratifying patients by age, and incorporating longer follow-up periods. Comparative studies with other manual therapy approaches, such as Mulligan or Kaltenborn, as well as multimodal rehabilitation programmes, would also be informative. In addition, cost-effectiveness analyses and studies assessing patient satisfaction could further support the integration of MAIT into clinical practice guidelines. Future studies should also investigate whether patient-specific factors, such as baseline symptom duration, physical activity levels, or occupational demands, modify the response to MAIT, thereby helping identify subgroups most likely to benefit from this intervention. Additionally, examining the neurophysiological mechanisms underlying MAIT through imaging, electromyographic, or biomechanical approaches could provide valuable insight into its therapeutic effects and inform more targeted clinical applications.

Overall, MAIT demonstrated superior improvements in pain, function, and ROM compared with KINE in patients with non-traumatic shoulder pathology. Because of its comparable workload for patients and physiotherapists and its favourable clinical profile, MAIT should be considered within clinical decision-making. These findings not only contribute to the growing body of evidence supporting manual therapy but also underline the need for future trials to consolidate Mailand’s role within shoulder rehabilitation programmes.
